# Quantitative molecular bioluminescence tomography

**DOI:** 10.1117/1.JBO.27.6.066004

**Published:** 2022-06-20

**Authors:** Alexander Bentley, Xiangkun Xu, Zijian Deng, Jonathan E. Rowe, Ken Kang-Hsin Wang, Hamid Dehghani

**Affiliations:** a University of Birmingham, School of Computer Science, College of Engineering and Physical Sciences, Birmingham, United Kingdom; b University of Birmingham, College of Engineering and Physical Sciences, Physical Sciences for Health Doctoral Training Centre, Birmingham, United Kingdom; c University of Texas Southwestern Medical Center, Biomedical Imaging and Radiation Technology Laboratory, Department of Radiation Oncology, Dallas, Texas, United States; d Johns Hopkins University, Department of Radiation Oncology and Molecular Radiation Sciences, Baltimore, Maryland, United States

**Keywords:** bioluminescence imaging, bioluminescence tomography, diffuse optical imaging

## Abstract

**Significance:**

Bioluminescence imaging and tomography (BLT) are used to study biologically relevant activity, typically within a mouse model. A major limitation is that the underlying optical properties of the volume are unknown, leading to the use of a “best” estimate approach often compromising quantitative accuracy.

**Aim:**

An optimization algorithm is presented that localizes the spatial distribution of bioluminescence by simultaneously recovering the optical properties and location of bioluminescence source from the same set of surface measurements.

**Approach:**

Measured data, using implanted self-illuminating sources as well as an orthotopic glioblastoma mouse model, are employed to recover three-dimensional spatial distribution of the bioluminescence source using a multi-parameter optimization algorithm.

**Results:**

The proposed algorithm is able to recover the size and location of the bioluminescence source while accounting for tissue attenuation. Localization accuracies of <1  mm are obtained in all cases, which is similar if not better than current “gold standard” methods that predict optical properties using a different imaging modality.

**Conclusions:**

Application of this approach, using *in-vivo* experimental data has shown that quantitative BLT is possible without the need for any prior knowledge about optical parameters, paving the way toward quantitative molecular imaging of exogenous and indigenous biological tumor functionality.

## 1 Introduction

Bioluminescent tomography (BLT) is a preclinical imaging technique used to recover the three-dimensional (3D) spatial and intensity distribution of bioluminescent molecules using two-dimensional (2D) images [bioluminescent imaging (BLI)] of light at the surface of an animal.^1^ This is achieved through computational optimization algorithms that incorporate models of the light transport through tissue, typically using the diffusion approximation of the radiative transport equation.^2^ A number of issues arise when carrying out BLT, which has to date, limited its preclinical use to surface weighted 2D BLI, with progress toward true tomography made over the last decade. A major challenge in BLT is nonuniqueness,^3^ which is addressed through the use of multispectral data, increasing the experimental time needed, as data is sequentially collected using filters.^4^ This has been addressed using, e.g., a hyperspectral system^5^ based on compressive sensing, by utilizing random projections data as captured using a spectrometer.

Often overlooked is the importance of having accurate computational models for the structure and the underlying optical parameters of the animal/tissue being imaged to allow accurate three-dimensional (3D) imaging through optimization.^6^ Structural knowledge can be gained through secondary modalities, such as MRI,^7^ CT,^8^ and ultrasound,^9^ which can be used to develop an accurate model for optimization and 3D reconstruction, although often relying on estimated tissue optical parameters. Heterogeneous models have been used^6^^,^^10^^,^^11^ to offer an alternative, through registration with an atlas model, or by defining permissible regions to improve accuracy.^12^^,^^13^ Work targeted toward direct imaging of optical properties through the use of diffuse optical tomography (DOT) have demonstrated promise, however, with increased imaging and development cost.^4^

Recent reconstruction algorithms based on machine learning and deep learning have shown great promise in diffuse optics-based applications,^14^ such as the detection of breast cancer using MRI-guided near-infrared spectral tomography.^15^ In BLT, e.g., Bayesian learning method based on K-nearest neighbor strategy, which is able to incorporate several types of a priori information, including anatomical information, has shown good accuracy for tumor spatial positioning and morphology reconstruction.^16^ A major challenge in the application of neural networks in BLT is the available training data sets that rely not only on the unknown bioluminescence source location but also on the unknown underlying optical properties, making it difficult to reconstruct the patterns outside the training data sets. A recent self-training strategy has been proposed for BLT where large-scale data can be generated with random target numbers, shapes, and sizes through a random seed growth algorithm allowing the neural network to automatically self-train, which is showing great promise for application in small animal studies.^17^

BLT in areas of research such as oncology has shown to be of benefit as a tool allowing high-contrast image-guided radiation therapy.^18^^,^^19^ Specifically, to advance image-guided irradiation for soft tissue targeting and treatment assessment, a unique multimodal system has been developed combining BLT with x-ray cone-beam CT (CBCT) for preclinical radiation research. The system has been verified using an orthotopic glioblastoma (GBM) model^18^^,^^20^ and orthotopic pancreatic ductal adenocarcinoma (PDAC) model,^21^ with accuracies of tumor location recovery of ∼1 and 2 mm, respectively. Limitations, however, exist from the need for a trial-and-error approach for optimizing the unknown optical parameters of the tissue, based on structural *a-priori* knowledge from the CBCT.

A novel optimization algorithm allowing simultaneous recovery of optical parameters directly from bioluminescence data has recently been proposed,^22^ but never applied, to biological data. Here, the first-ever *in-vivo* application of an optimization algorithm, recovering both the bioluminescence source and intensity as well as the tissue absorption (total hemoglobin content) in small animal BLT system is presented.

The challenges and limitations as observed in commercial preclinical BLI/BLT systems will be highlighted, using the example models of orthotopic GBM and self-luminous light source implanted in pancreas to highlight the benefits of the proposed algorithm. Not only is it possible to accurately recover the spatial distribution of a bioluminescence source, but also the potential to recover biologically informative parameters such as hemoglobin content, without the need for additional assumptions, allowing quantitative BLT.

## 2 Materials and Methods

### 2.1 Animal Welfare

All animal experimental procedures were carried out in accordance with the Johns Hopkins University Animal Care and Use Committee under strict accordance with institutional guidelines and regulations for the use of vertebrate animals.

### 2.2 Orthotopic GBM Mouse Model

To establish the orthotopic GBM mouse model, a C57BL/6J mouse (female, 8-weeks-old; Jackson Laboratory, Bar Harbor, Maine, United Stated) was immobilized on a stereotaxic instrument (Catalog No. 51730; Stoelting, Wood Dale, Illinois, United States) at prone position. A 5- to 8-mm sagittal incision on the scalp was made. A 0.5-mm-diameter parietal burr hole at 20-mm anterior to the lambdoid suture and 2-mm left to the sagittal suture of the skull was made with an electric bone drill (Catalog No. 51449; Stoelting, Wood Dale, Illinois, United States). $1.2\times 10^{5}$ GL261-*Luc*2 cells in 2  μL of phosphate-buffered saline (PBS) were injected 3-mm deep into the mice brain by utilizing a 10-μL Hamilton gas-tight syringe with 32-gauge blunt-tip needle (Catalog No. 53485-1; Stoelting, Wood Dale, Illinois, United States). 1  μL of cell suspension was injected and after waiting for 1 min these two steps were repeated until all suspension was injected. After the injection was complete, the syringe was raised 1 mm and after 1 min wait these two steps were repeated until the needle was completely moved out of mouse head. The skull opening and incision were sealed with tissue adhesive (No. 1469SB; 3M Vetbond^TM/MC^, St. Paul, Minnesota, United States).

### 2.3 Implantation of Self-Luminous Light Source into Mouse Pancreas

A self-luminous light source (cylinder, 0.9 mm in diameter, 2 mm in length) was surgically implanted into the pancreas of albino C57BL/6J mouse (female, 8-weeks-old; Jackson Laboratory, Bar Harbor, Maine, United States). The surgical site was located at the skin on the mouse left flank, around spleen position, *posterior* to rib. An 8- to 12-mm transverse incision was made on the skin at the surgical site, and another 8- to 12-mm transverse incision was made on the peritoneum right under the skin incision. The spleen and pancreas were gently exteriorized, and the pancreas was spread out on the skin. A small pocket was created in the pancreatic parenchyma, and the light source was inserted into the tissue pocket. A 7-0 suture (CC1107N-45; AROSurgical™, Newport Beach, California, United States) was used to close the tissue pocket of the pancreatic parenchyma. After placing the pancreas and spleen back into the abdominal cavity, the incisions on the peritoneum and skin were closed using a 4-0 suture (E15A04L-45; AROSurgical™, Newport Beach, California, United States).

### 2.4 BLT System

Two BLT systems in a similar configuration and equal performance were utilized in this study. The first one is an in-house BLT system^18^ consisting of an optical assembly, a thermostatic system, a transportable mouse bed, and a mobile cart. The optical assembly is driven by a motorized linear stage to dock onto the mouse bed for imaging. The assembly contains a rotatable three-mirror system (98% reflective, protected silver coating) with four light-emitting diodes mounted at its corners for photo imaging, a filter wheel (Edmund Optics Inc., Barrington, New Jersey, United States), a charge-coupled device (CCD, iKon-L936; Andor Technology, Belfast, United Kingdom) mounted with a 50-mm f/1.2 lens (Nikkor, Nikon Inc., Melville, New York, United States), and a light-tight enclosure. The optical signal emitted from an imaged object was directed by the three-mirror system, passing through the filter wheel and captured by the CCD camera. The image taken at top of the mouse bed is labeled as 0-deg projection imaging. The three-mirror system can rotate 180 deg (from −90  deg to 90 deg) around the imaged object for multiprojection imaging. The focal plane was set at ∼11  mm above the mouse bed with the three-mirror system placed at 0 deg. The pixel scale is the corresponding physical size of CCD pixel at focal plane, which is 0.12 mm per CCD pixel. The optical path from the focal plane to the front surface of the camera lens is 45 cm. Four 20-nm full-width-half-maximum (FWHM) bandpass filters (Chroma Technology Corp., Bellows Falls, Vermont, United States) at 590, 610, 630, and 650 nm were mounted in the filter wheel for multispectral imaging. The thermostatic system, which is built in the light-tight enclosure (except for the heat gun) and consists of a resistor, a thermocouple with monitor, seven fans, and a heat gun with heat transport pipeline linked to the enclosure which was used to boost and maintain the temperature around the imaged object at 37°C. The transportable mouse bed allows the imaged object transferred from the optical system to CBCT system for CBCT imaging. Eight ball bearings (BBs; PTFE balls, 2.4-mm diameter; McMaster-Carr, Santa Fe Springs, California, United States), which can be identified both in optical photo image and in CBCT image, were attached on the bed as the fiducial markers for registering the coordinates of optical and CBCT system. The *in vivo* data of GBM-bearing mouse were acquired by the in-house BLT system.

Our team and our industrial partner Xstrahl Inc. developed the second BLT system, a commercial configuration MuriGlo (Xstrahl Inc., Suwanee, Georgia, United States), built from the same concept of the in-house system with improved capability of acquiring full 360-deg optical projection. The 360-deg imaging acquisition is especially useful for abdominal imaging, such as pancreas site, to obtain all the essential projections for BLT reconstruction. Optical signal emitted from the animal is reflected from a three-mirror system to a fixed 45-deg mirror, passing through filter and captured by a CCD camera (iKon-M 934; Andor Technology, Belfast, United Kingdom). The three-mirror system supports the 360 deg multiple projection imaging. The fixed mirror and iKon-M934 CCD allow for a compact configuration. The focal plane was set at ∼16  mm above the mouse bed with the three-mirror system placed at 0 deg, and the pixel scale is 0.10 mm per CCD pixel. The optical path from the focal plane to the front surface of the lens is 40 cm. The *in-vivo* data of the mice implanted with light source in pancreas was acquired by the MuriGlo system. Details of both systems utilized in this work, together with system characterization and evaluation have been outlined previously.^18^^,^^21^^,^^23^

### 2.5 Quantification of System-Specific Cell Spectrum

Because of the multispectral BLT approach, it is important to quantify the optical system spectral response and the emission spectrum of bioluminescent tumor cells. The measurement via our optical systems includes spectral responses of the system and cell, and the resulted spectrum is referred to as the system-specific cell spectrum. Therefore, the wavelength dependent BLIs can be normalized to the measured spectrum weighting, used as the input data for the BLT reconstruction.

The system-specific spectral weights of GL261-*Luc2* cells at 590, 610, 630, and 650 nm in petri dishes with cells >80% confluency at concentration of 0.75 mg of D-Luciferin per 1 mL of PBS at 37°C as set by the in-house optical system were measured. To eliminate the change of the *in vitro* spectral signal as function of luciferin incubation time, open field images without filters were taken before and after each spectral BLI to quantify the *in vitro* signal variation over time. The time point for each image was recorded and the open field images were used to generate an *in vitro* time-resolved signal curve. The intensity of multispectral BLIs was corrected based on time-variant data from the time-resolved curve. The measured spectrum of the GL261-*Luc2* at 590, 610, 630, and 650 nm are 1, 0.916±0.014, 0.674±0.019, 0.389±0.012 (n=20), respectively. The same procedure was applied to measure the spectrum of the self-luminous light sources. At 590, 610, 630, and 650 nm, the spectrum weights for the light sources in the mice [as shown in Figs. 7(a) and 7(b)] are 0.942, 1, 0.893, 0.633, and 0.949, 1, 0.886, 0.626, respectively. The spectral response of the in-house and MuriGlo system was also investigated and as the difference is insignificant, the measured spectrum was applied to both systems.

### 2.6 *In Vivo* Bioluminescence Imaging

The GBM-bearing mouse was subject to multiprojection and multispectral bioluminescence imaging with the in-house optical system two weeks after the cell implantation. In preparation for bioluminescence imaging session for GBM-bearing mouse, mouse hair was shaved with a clipper, followed by hair depilation. D-Luciferin (125  μL, 30  mg/mL for 25 g mouse to reach 150  mg/kg, XenoLight D-Luciferin $K^{+}$ Salt, PerkinElmer Inc., Waltham, Massachusetts, United States) was administrated via intraperitoneal injection. BL imaging was conducted 10 min after the D-Luciferin injection. Mouse was anesthetized with 1% to 2% isoflurane (Fluriso, MWI Veterinary Supply Co. Boise, Idaho, United States) in oxygen while imaging. Multiprojection (0 deg, 90 deg, and −90  deg) and multispectral (590, 610, 630, and 650 nm) BLIs were acquired at 8×8 binning (0.96  mm/pixel at imaging plane), 4× preamplifier gain, and 1 MHz readout rate. Because the *in vivo* signal at 590 nm was weak compared with those at other wavelengths, which would affect the stability of the BLT reconstruction, the images at 610, 630, and 650 nm were only used for this study. Open field images were acquired before and after each spectral image to build time-resolved curve for *in vivo* bioluminescence signal among different projections,^18^ which was used to quantify the bioluminescence signal change during the bioluminescence imaging course.

The mouse bearing self-luminous light source in pancreas was subject to multiprojection and multispectral BLI (8×8 binning, 0.8  mm/pixel at imaging plane) in MuriGlo one week after the light source was implanted. The procedures of BLI acquisition for the pancreatic light source study were similar to those used for the GBM study. Since the implanted light source is closer to the surface of abdomen than that of dorsum, the supine position was chosen for the BLI acquisition. Due to the location of light source, no or minimum signal was detected at 90-deg and 180-deg projections, only the 0 deg and −90  deg projections were chosen as the surface input data for the BLT reconstruction. Images at multiple projections were taken to retrieve the BB positions on mouse bed for mapping the BLI onto 3D mesh surface generated from the CBCT imaging as the input data for BLT reconstruction.

### 2.7 CBCT Imaging

After the bioluminescence imaging, the mouse bed with an animal was transferred from the optical system to the small animal radiation research platform (SARRP; Xstrahl Inc., Suwanee, Georgia, United States) designed for CBCT-guided irradiation study.^18^ CBCT image was acquired by rotating the animal with a 4D robotic-controlled bed (3-axis translation and 360-deg rotation) between an amorphous silicon flat-panel detector and an x-ray source at 0.4-mm focal spot, 65 kVp and 0.7 mA with 1-mm-thick aluminum filter. Studied animal was anesthetized with 1% to 2% isoflurane in oxygen during the animal transportation and imaging course. The acquired SARRP CBCT image was used to provide anatomical structure of animals to generate a computational model (tetrahedral mesh) for the BLT reconstruction. For the pancreatic mouse model, the mesh consisted of 43,580 nodes corresponding to 237,110 linear tetrahedral elements, whereas for the GBM model it consisted of 6294 corresponding to 32,704 linear tetrahedral elements.

Contrast CBCT was used to assess the actual volume of the *in vivo* GBM,^18^ and the center of mass of the contrast-labeled tumor volume was calculated for BLT localization assessment. An in-house high-resolution CBCT system,^24^ providing superior image contrast than that of SARRP CBCT, was employed to identify the contrast-labelled GBM. The mouse was imaged 1 min after the contrast injection at dose of 2  gI/kg (Iodixanol, retro-orbital injection at 320 mgI/mL, Visipaque, GE Health Care, Chicago, Illinois, United States).

### 2.8 Data Mapping for Multiprojection BLIs

Because SARRP CBCT imaging defines the coordinate used for BLT reconstruction, our geometry calibration method was published in Refs. 18 and 25 were used to map the 2D BLIs acquired at different projections onto the discretized mesh surface of the animal CBCT image. The mapped BLIs were used as the input data for BLT reconstruction. The method has two steps: (1) mapping the CBCT coordinate to the 3D optical coordinate with rigid transformation and then (2) projecting the 3D optical coordinate to the 2D optical (CCD) imaging plane. After the 3D CBCT and 2D optical coordinates are registered, for a given projection, the surface BLI can then be mapped to the CBCT image. The data mapping process requires knowledge of the geometrical parameters of optical system. The BBs on the mouse bed can be located in both CBCT and 2D optical images. An optimization routine with the constrained multivariable optimization function (fmincon, MATLAB, The MathWork Inc., Natick, Massachusetts, United States) was developed to retrieve the geometrical parameters by minimizing the difference between the calculated and measured BB positions in the 2D optical coordinate; the BB positions in 2D images were used as the measured positions, and the corresponding BB positions retrieved from the optimization routine based on the optimized geometrical parameters and the BB positions in 3D CBCT were used as the 2D calculated positions. The geometric calibration was performed for each animal imaging session to ensure accurate data mapping for BLT reconstruction.

The BLIs were then mapped onto the 3D mesh surface of the imaged mouse generated from the CBCT image. At the overlapped region on the mesh surface, for a given node between two mapped images from different projections, the maximum value of the two images was chosen as the value on that surface node. The mapped surface data larger than 10% of the maximum value among all the surface points were used as input data for BLT reconstruction.

### 2.9 Algorithm

The final algorithm needed to complete the entire framework is one that calculates both the optical properties, given the surface fluence rate data at multiple wavelengths, as well as the location of the source within the subject. To resolve unknown optical properties, DOT is a commonly used imaging technique, known as named due to the fact that the transport of light through tissue at wavelengths in the visible and near-infrared (NIR) bands become nearly isotropic, therefore is well defined by photon diffusion. The software package, NIR fluorescence and spectral tomography (NIRFAST), was utilized to allow for the simulation of light propagation within biological tissue using a finite element method (FEM), documented elsewhere.^2^ Within the package are a number of forward and inverse models, and a spectrally constrained case is utilized in this work.^26^ The algorithm has been modified to take the estimated internal bioluminescence source location and surface fluence data with the goal of directly estimating the concentrations of total hemoglobin (cTHb) as well as scattering power and amplitude,^26^ where cTHb is the sum of oxy-hemoglobin and deoxy-hemoglobin. The assigned chromophore concentrations can be used to calculate the underlying wavelength dependent absorption coefficient of tissue through the use of extinction coefficients of individual chromophores. This can similarly be done using the scattering power and amplitude to obtain a corresponding reduced scattering coefficient at each wavelength using Mie scattering theory. Using a continuous wave model, the Jacobian (function to allow mapping of measured data to internal optical properties) is calculated and then inverted using the Moore–Penrose generalized inverse, which is typically more suitable toward underdetermined problems.^27^ The Moore–Penrose generalized inverse finds the “best fit” or minimum norm solution to a system of linear equations, the implementation of which has been extensively detailed elsewhere.^2^ From this an update in optical properties is calculated and the whole process is repeated, new boundary data is simulated and compared with the original data to calculate a projection error. This error is then used as a stopping mechanism for when it is considered that convergence has occurred, typically within 2% change. Regularization is used alongside the Moore–Penrose generalized inverse with a starting value of 0.1, which is the standard value used as detailed elsewhere.^2^ All of the reconstruction parameters used for all the data presented are consistent across all model, with the only variable being the initial estimate of the optical properties.

Algorithm 1 outlines the steps for image reconstruction and is briefly summarized: First, multispectral data are collected as described already, which are used with an initial “estimate” of underlying optical properties to perform tomographic reconstruction of the spatial bioluminescence light distribution as achieved using a compressive sensing conjugate gradient (CSCGNW) algorithm described elsewhere.^29^ The center of mass of the reconstructed bioluminescence source is then defined as the “source position” and using the DOT algorithm and the same dataset, the optical properties of the medium are calculated. From this, if the error conditions have not yet been met, the optical properties are updated by the average value across the entire model or of a particular predefined volume within. Using these new updated optical properties, the CSCGNW algorithm is then used again to reconstruct the new spatial bioluminescence light distribution and whole process is repeated iteratively. As with the inverse problem outlined already, a stopping condition is put in place to stop the algorithm when it is considered to have converged. The stopping criteria is the same as that used in the DOT algorithm whereby a projection error is calculated between the original and modelled data using the updated optical properties. The iterative process is continued until the change in projection error is below a tolerance which is typically set to 2%. When modeling the new source location for the update of optical parameters using the DOT algorithm, first the reconstructed bioluminescence source size and location is represented as its FWHM and the distributed source (i.e., uniform intensity) at the recovered FWHM is then set as the new update.

**Algorithm 1:** Outline of image reconstruction

• Set total hemoglobin concentration and scattering parameters (μ)
• Define termination criterion
• **Repeat**
• Reconstruct the spatial distribution of Bioluminescence source using boundary data using spectral derivative method^28^
• Define “diffuse source” as all nodes >= full-width-half-max of reconstructed bioluminescence source
• Calculate center of mass of diffuse source, S
• Set location of diffuse source as S
• Calculate the Jacobian for diffuse source and all boundary data using the adjoint method^2^
• Update μ using the Jacobian and boundary data
• Calculate project error, χ
• **Until** ‖χ‖ < termination criterion

## 3 Results and Discussions

Traditional biological preclinical studies that utilize bioluminescence imaging (BLI) typically only use topographic data that is captured at the surface of an animal, taken at a determined wavelength and view. This method allows for qualitative information about the underlying source to be inferred from the intensity of light measured at the surface. Issues arise when employing this method due to the spectral dependence on absorption and scattering of light traveling through diffuse biological tissue. This implies that the intensity of light measured at the surface of the animal will be different depending on the detected wavelength. Another issue arises depending on the viewpoint of the animal used when imaging takes place whereby the maximum and total intensity measured will change depending on the viewpoint used because light propagation in space being dependent on the viewing angle.

**Fig. 1:**
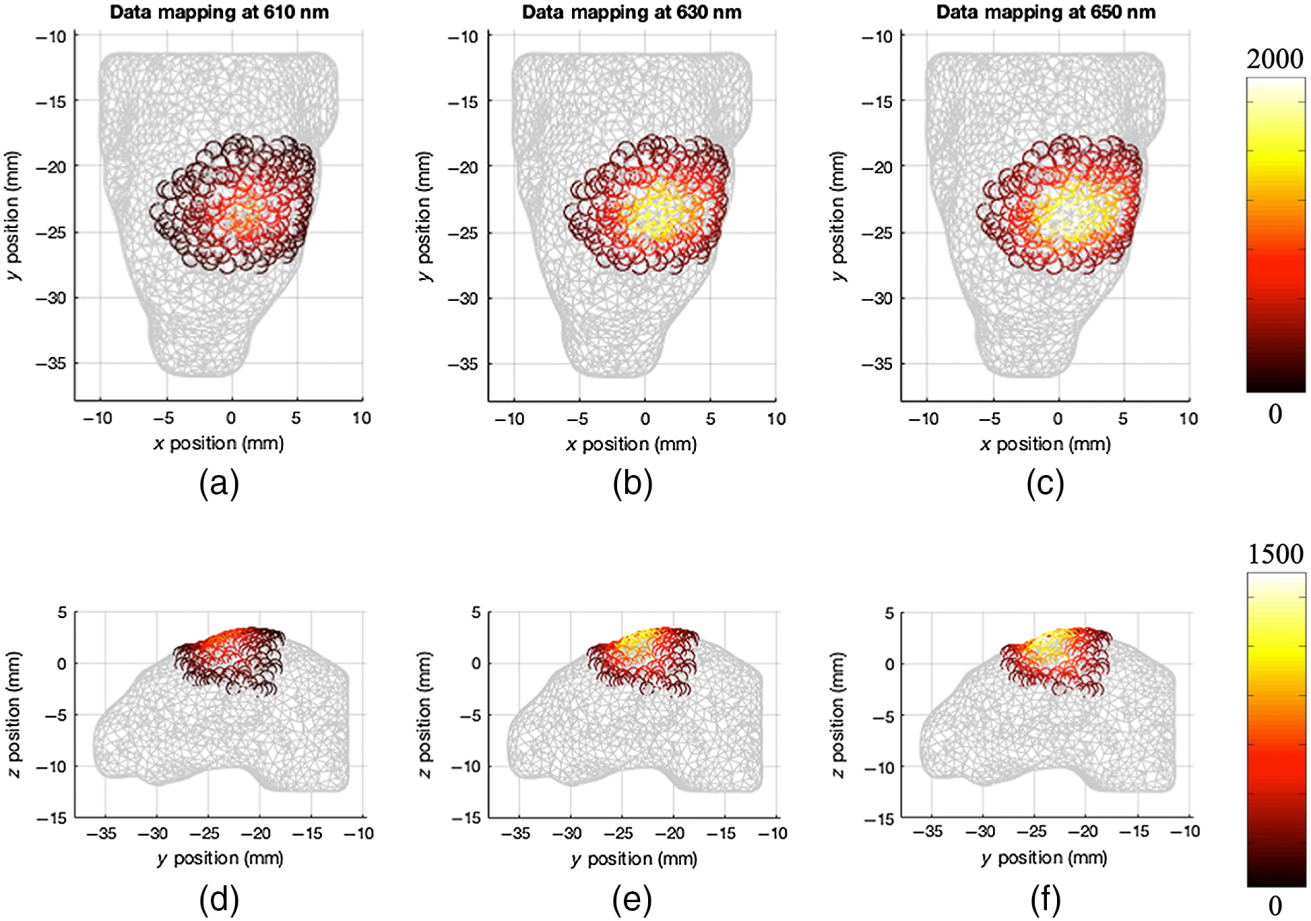
Top and side topographic views of bioluminescence signal for GBM model at 610, 630, and 650 nm. (a)–(c) Top views and (d)–(f) side views.

**Fig. 2:**
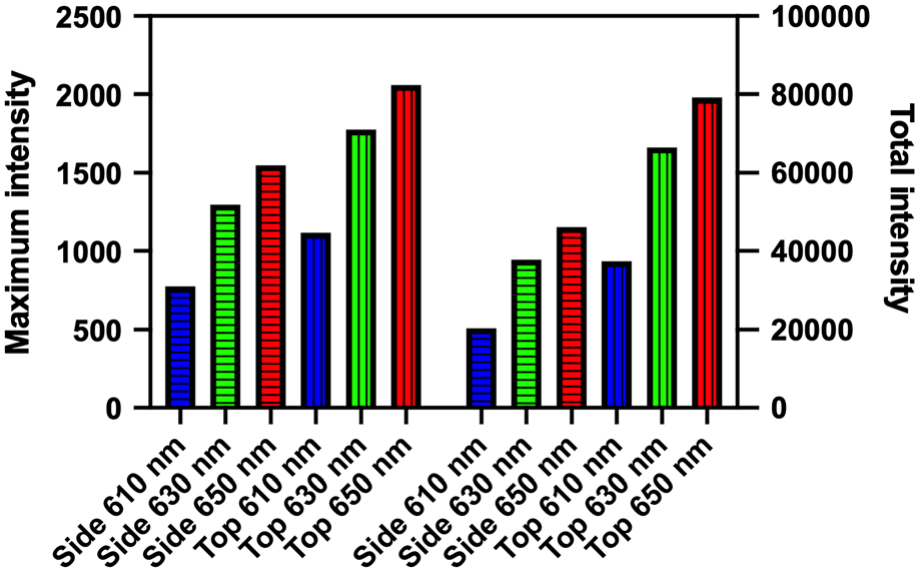
Effect of using topographic bioluminescence images for quantification at different wavelengths and views for (left) maximum intensity and (right) total intensity.

These effects are visualized in Figs. 1 and 2, whereby surface fluence data has been measured from the orthotopic GBM model outlined above, using three wavelengths, 610, 630, and 650 nm. The top and side views of the surface fluence at each wavelength are shown in Fig. 1, where the difference in measured intensity in each case is apparent. These differences are quantified using the bar chart shown in Fig. 2, where the total and maximum intensity for each viewpoint and wavelength are shown.

**Fig. 3:**
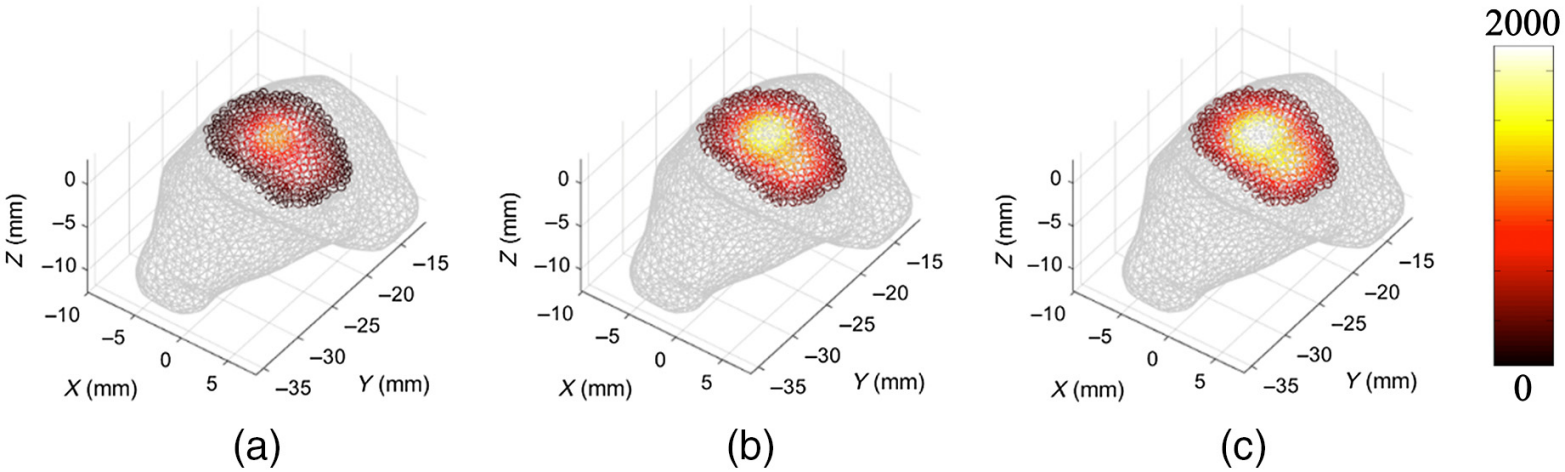
3D surface fluence signal for GBM model.

Because of the spectral and viewpoint dependency of light intensity measured when collecting 2D topological fluence data, it is impossible to confidently infer the underlying light source distribution. To counter this, through the collection of multiple viewpoints, it is possible to build a 3D topological image of the light intensity at the surface of the animal. This provides the ability to infer the underlying light distribution with more confidence, however, images still suffer from the spectral variation of absorption and scattering properties found in biological tissue and free-space light propagation. Figure 3 shows the same GBM data as before, however as a 3D topological image. There are computational methods available that can be used to correct these images for the spectral differences and emission of the bioluminescent source used, however, it is still difficult to use the surface data alone to make any significant assumption about the size or location of the source. This is due to nonuniqueness, meaning that a shallow weak source may give the same intensity measurements as a deeper more intense source.

BLT takes BLI a step further, by using prior knowledge of the animal being imaged to reconstruct the 3D spatial distribution of bioluminescent molecules located within the animal using model-based data optimization. To do this a number of conditions need to be satisfied, the first being the need for an accurate computation model of the animal. Additionally, traditional BLT requires accurate knowledge of the underlying optical properties of the animal being imaged. Furthermore, in order to address the issue of nonuniqueness, it is necessary to collect surface fluence data at multiple wavelengths,^3^ due to the spectral dependence of the optical properties. Finally, when carrying out tomographic model-based reconstructions, it is common to use the raw intensity of light measured at each wavelength that has been corrected for the bioluminescent emission spectrum of the molecules. Issues arise from doing this, which relate to the position and shape of the animal being imaged, due to the Lambertian nature of light exiting a boundary of diffuse media. To account for this, it is possible to use a free-space model,^30^ however this can be time-consuming and complex. To overcome this problem, it was shown that by using spectral derivative data, which utilizes the “logarithm of intensity,” it is possible to improve the quantitative error of BLT from 49% to 4%, without the need to collect additional data or make any modifications to existing imaging systems,^28^ which has been utilized in this work.

**Fig. 4:**
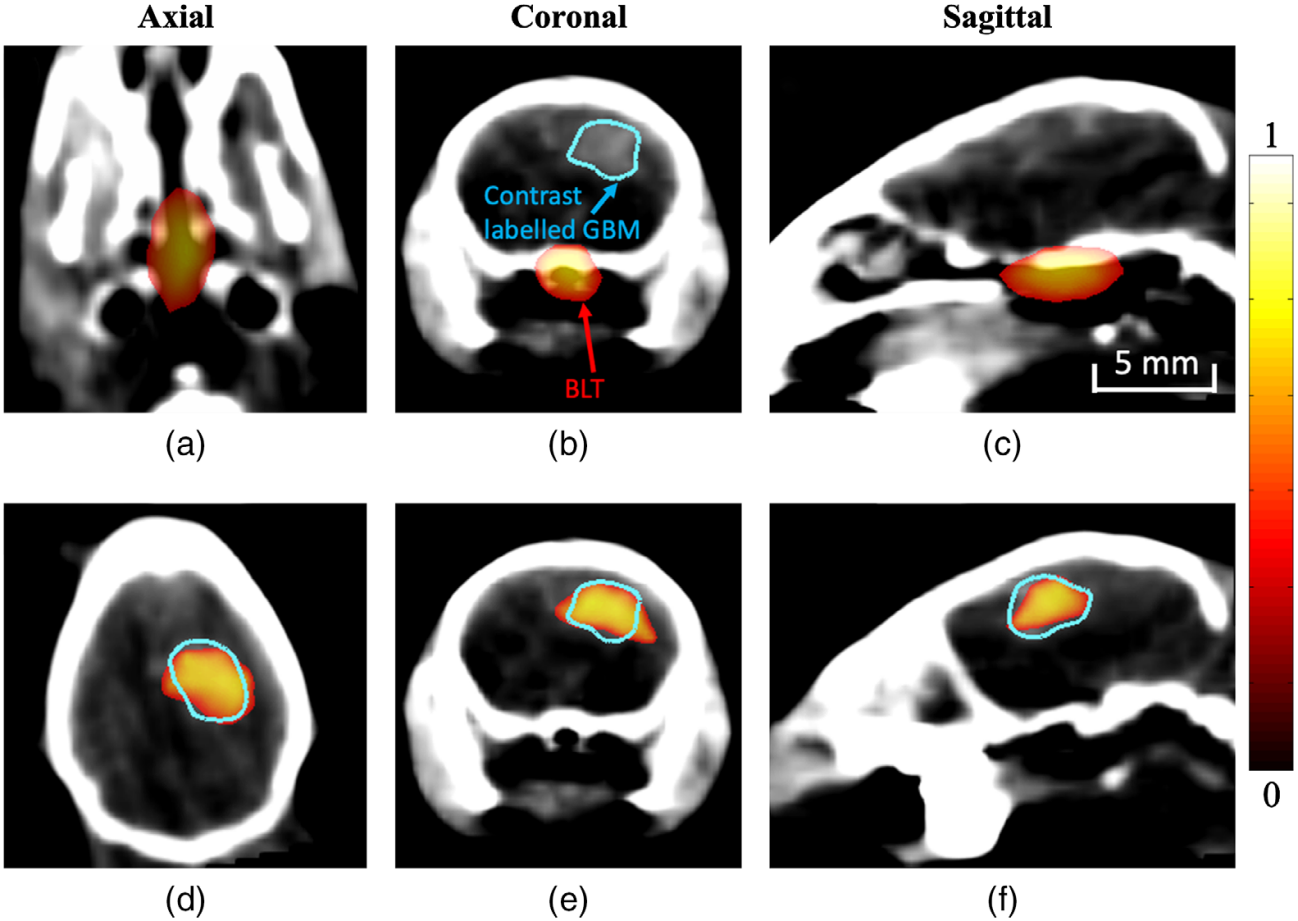
3D tomographic reconstructions (normalized) of bioluminescence GBM model using (a)–(c) optical parameters using an underestimated assumption (0.05 mM cTHb) and (d)–(f) using an overestimation of underlying optical parameters (0.2 mM cTHb). The blue contour shows contrast labeled GBM.

**Fig. 5:**
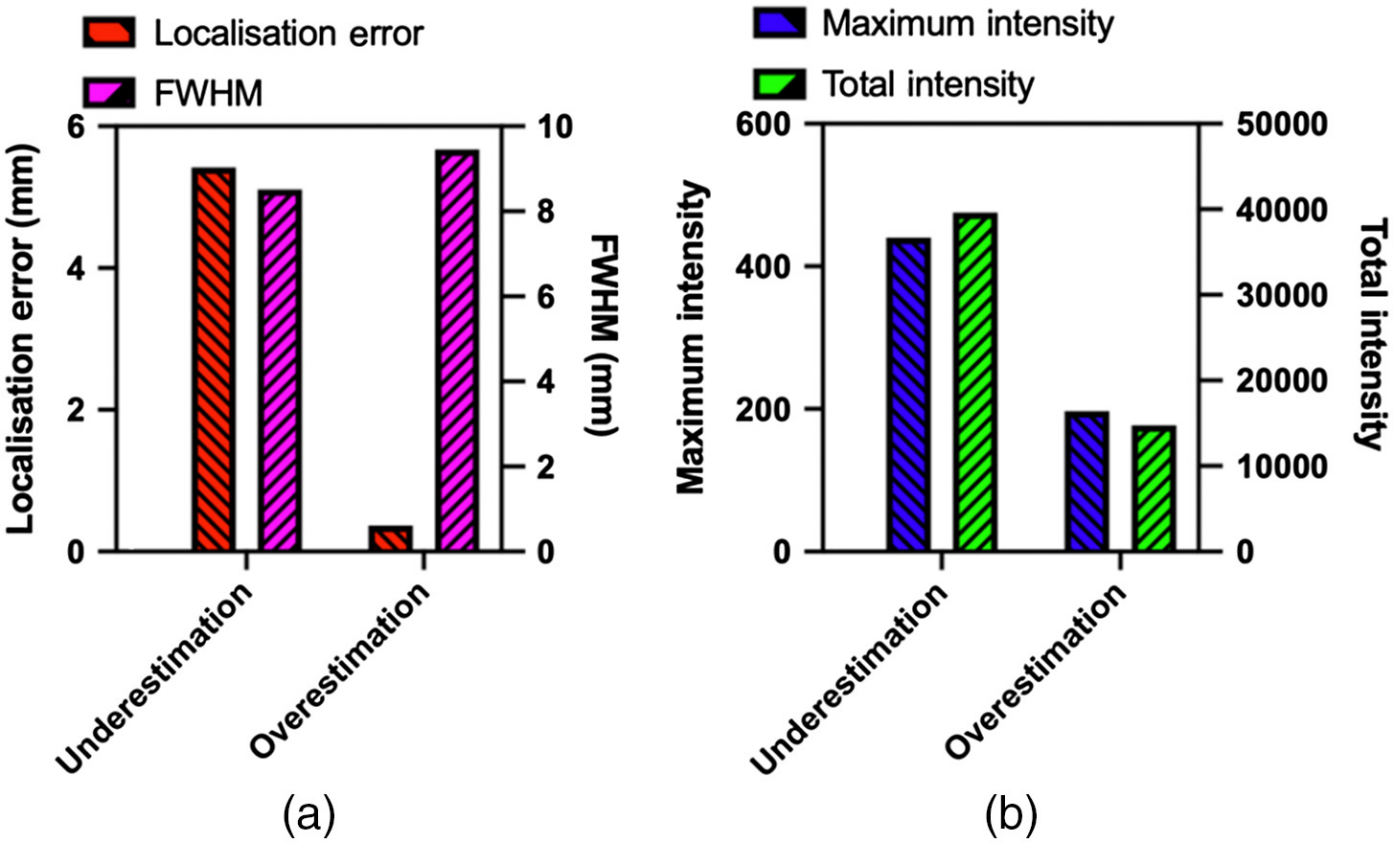
Calculated values of localization error, FWHM, maximum intensity, and total intensity based on differing assumptions of optical properties.

The importance of having accurate knowledge of the underlying optical properties to account for light propagation in model-based optimization is shown in Figs. 4 and 5. Figure 4 shows tomographic reconstructions using the surface fluence rate data of the same GBM model, using two different assumed concentrations of total hemoglobin (cTHb) as the optical properties used for model-based reconstruction. Reconstruction techniques are carried out using a spectral derivative method and are then thresholded to half the maximum value present.

Figures 4(a)–4(c) represent tomographic reconstruction when an underestimation of optical properties was used (cTHb=0.05  mM) with the results visually showing a larger, much deeper reconstruction. Figures 4(d)–4(f) represent tomographic reconstruction when an overestimation of optical properties was used (cTHb of 0.2 mM) with the results visually showing a larger and more superficial reconstruction. Differences in the reconstructions are quantified in Fig. 5 using four different metrics. The first metric used is localization error, which is the Euclidean distance between center-of-mass (COM) of reconstruction and ground truth location which was measured based on the contrast labeled GBM shown in the CBCT image. It can be seen that the localization errors of the two assumed optical properties are vastly different, clearly demonstrating the underlying effect of the assumed optical properties. The second metric used is the FWHM distance, which is the largest distance between reconstructed points at half the maximum value. As can be seen, both the under and overestimated reconstructions show an FWHM that is large, as visually evident in Fig. 4. The third and fourth metrics used are maximum and total reconstructed intensity. If using an underestimation of optical properties, it can be seen that the reconstruction has a maximum and total intensity that is larger than that of an overestimation of optical properties. All of these calculated metrics show the importance of using accurate optical properties when carrying out tomographic imaging, as they all represent significant values that are taken into account in biological studies.

To allow the use of BLT without the need to assume the underlying optical properties of the animal through the use of a second imaging modality, the algorithm as discussed above (Algorithm 1) used the knowledge of the emission spectrum of the bioluminescent source to employ an iterative method of optimizing optical properties whilst simultaneously localizing the source accurately. To show the benefit of applying this algorithm to real experimental data, it is used on the same GBM mouse model, above.

**Fig. 6:**
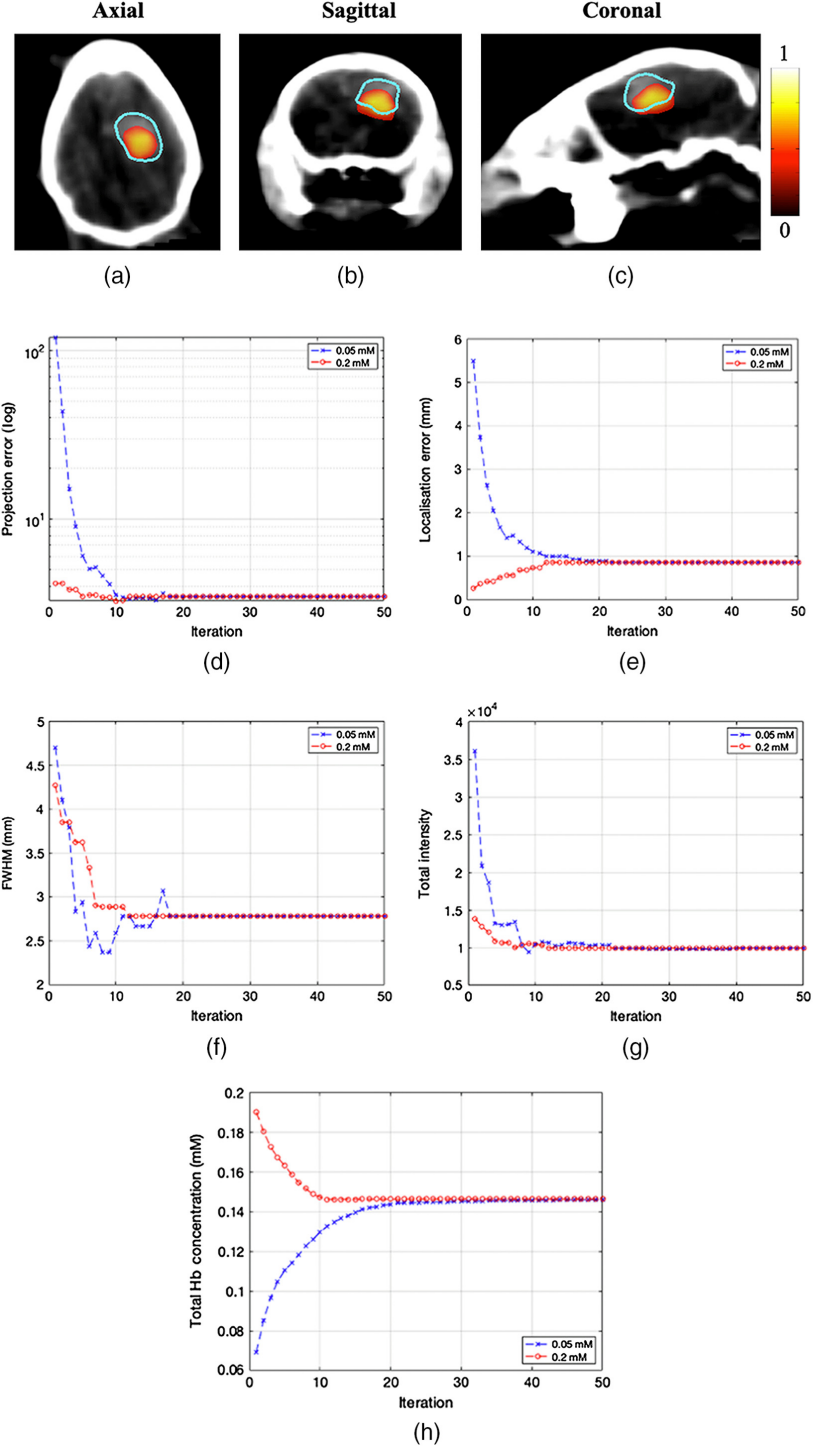
(a)–(c) Final reconstruction using the multivariant optimization algorithm. (d) Projection error versus iteration for low and high initial cTHb, (e) COM localization error, (f) FWHM distance, (g) total intensity, and (h) calculated cTHb of tissue.

The algorithm is initialized using starting “estimate” of cTHb of 0.05 and 0.2 mM, which represents the under and overestimates in Fig. 4. Figures 6(a)–6(c) show the final iteration of the algorithm when using either of the starting points. Figure 6(d) shows the projection error calculated at each iteration of the algorithm and it can be seen that both initial estimates converge to the same error. Figure 6(e) shows the calculated localization error of the tomographic reconstruction at each iteration and is shown that both starting points result in a localization error of ∼0.87  mm, which is similar if not better than values stated in literature using “gold” standard methods to obtain optical properties.^18^ Figures 6(f)–6(g) show the calculated FWHM and total intensity recovered at each iteration, which converge on 2.8 mm and 9868 counts, respectively. Finally, Fig. 6(h) shows the average concentration of total hemoglobin calculated at each iteration. This calculated cTHb converges to 0.145 mM assuming 70% oxygen saturation across the entire animal. Although the localization error and the FWHM can be quantitatively evaluated with respect to the contrast labeled GBM, it is challenging to evaluate the accuracy of the recovered total intensity and cTHb, but it is worth noting that using both initial estimates for the reconstruction it converges to the same value.

**Fig. 7:**
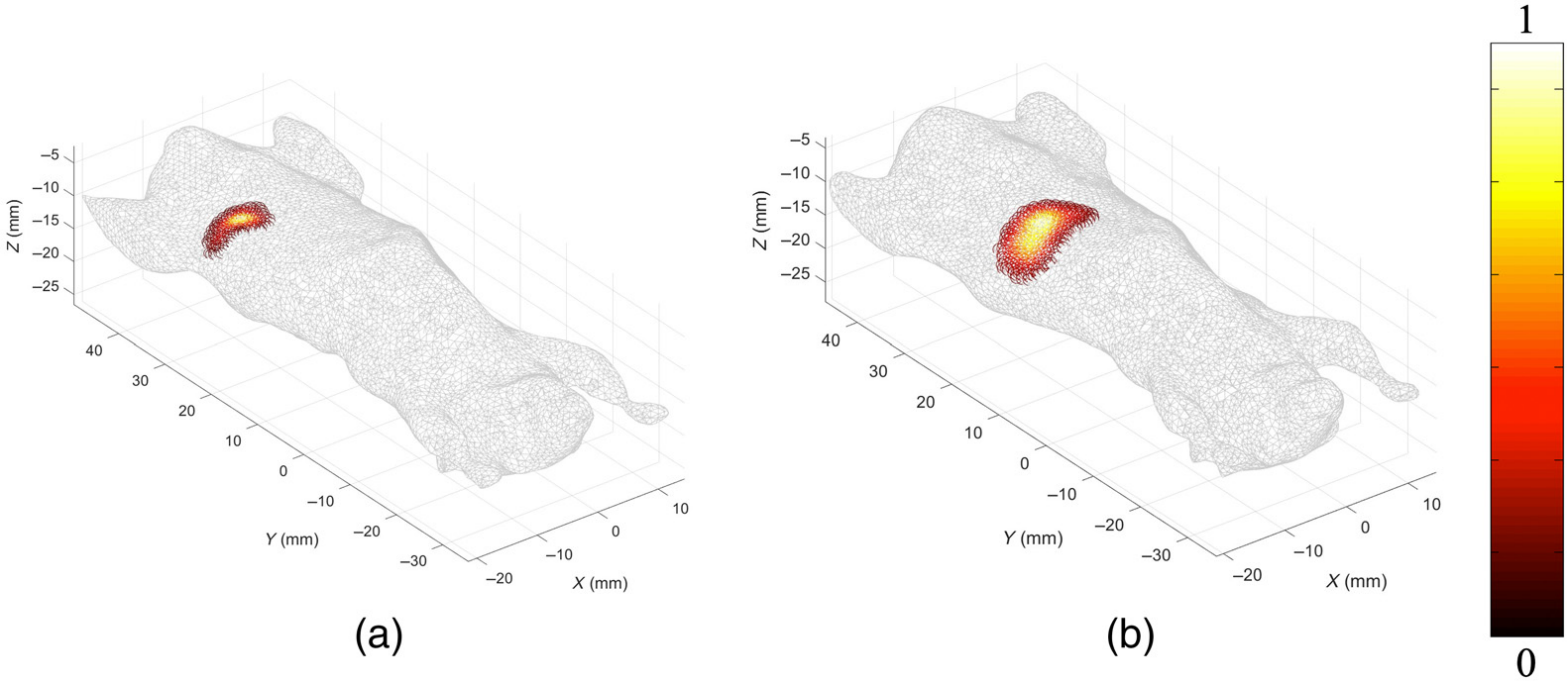
(a) and (b) Normalized 3D surface fluence rates at 650 nm for two mice with self-luminous light source implanted in pancreas.

To further, demonstrate the ability of the optimization algorithm on more complex models, and to evaluate its accuracy in recovering total intensity, it was next applied to two mice with a self-luminous light source implanted in the pancreas. The light sources are visible in CBCT image, and thus are used as the ground truth to assess accuracy. Furthermore, in both cases, as the same type of source is used, it is expected that the recovered total intensity to be similar, regardless of the animal model used. These mice differ from the GBM example such that the whole animal was imaged, resulting in a much larger reconstruction space (43,580 nodes) and hence greater numbers of unknowns. Figure 7 represents the normalized topographic images collected at 650 nm for each of the light source-implanted mice with each case the bioluminescent source being at a different depths and locations.

**Fig. 8:**
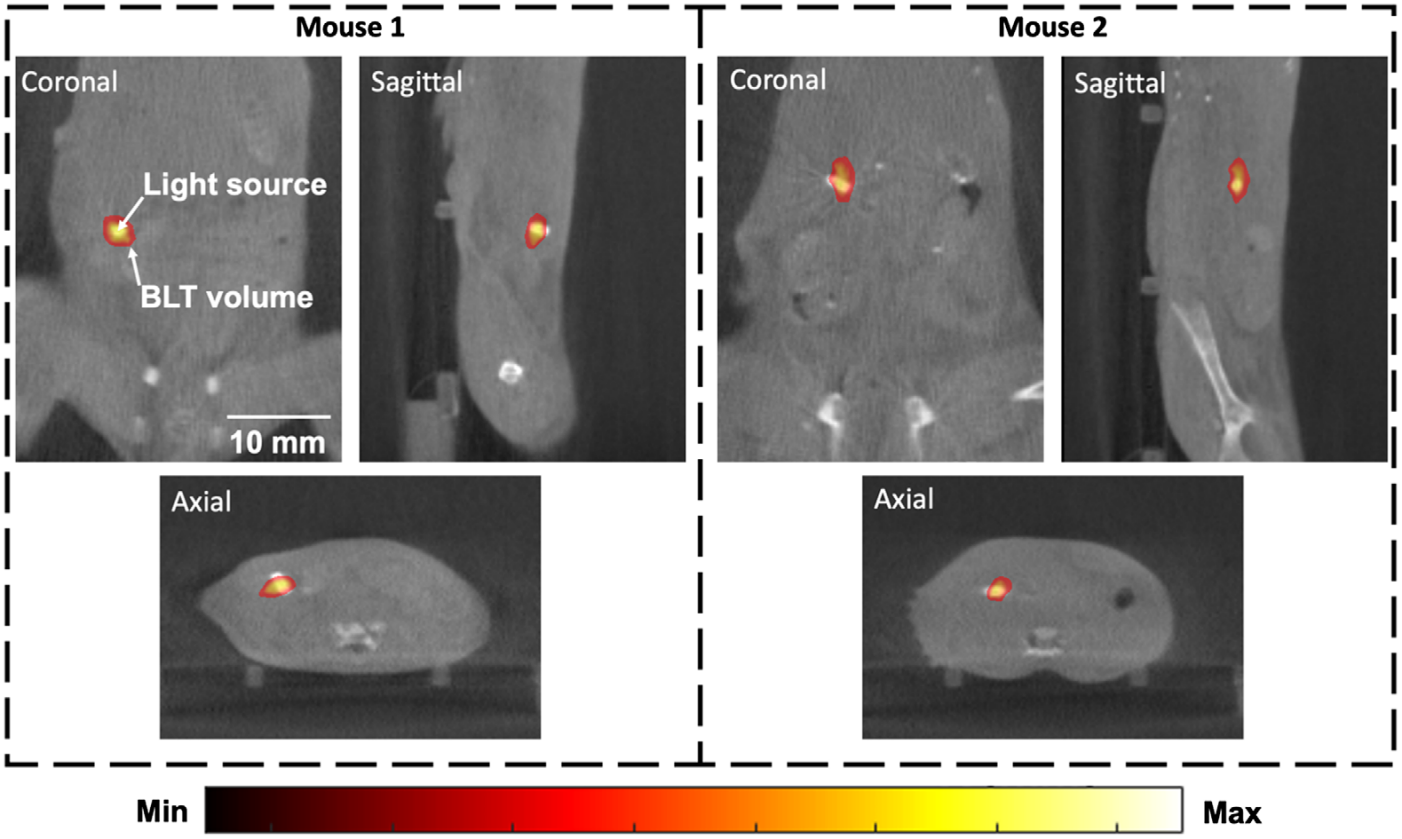
3D tomographic final iteration reconstructions for two mice with light source implanted in pancreas.

For each case, the surface fluence rate data was collected at three wavelengths (610, 630, and 650 nm) and then processed through the optimization algorithm using an initial “estimate” of 0.05 mM for cTHb. The algorithm ran for 50 iterations for each case and typically converged to a solution before all iterations were complete. Figure 8 displays the tomographic reconstructions for the final iteration of each mouse.

To quantify the efficiency and accuracy of the reconstructions, the same metrics as used for the GBM model were utilized and shown in Table 1. First, the localization error of each model was calculated, as compared to the ground truth location, which was found to be <0.7  mm over the two mice. The total and max intensities of each mouse were found to be ∼5400 and ∼285, respectively, and strikingly similar, demonstrating consistency in recovering source strength. The FWHM distance of the reconstructed sources across the two mice was <4.5  mm. The calculated concentration of total hemoglobin across both mice was found to be 0.1121 and 0.1151 mM.

**Table 1:** Reconstructed cTHb, COM localization error, total intensity, maximum intensity, and FWHM from the initial and final reconstructions for the mice with light source implanted in pancreas.

Mouse	THb concentration (mM)	Localization error (mm)	Total intensity (AU)	Max intensity (AU)	FWHM (mm)
1	0.1121	0.42	5461	287	3.8
2	0.1151	0.69	5327	286	4.5

## 4 Conclusions

This work highlights the progression and historical advancements made in BLI and BLT, to demonstrate the significance of a new proposed computational algorithm that accounts for the unknown optical attenuation of biological tissue. BLI measures light fluence rates from a bioluminescent source at the surface of the animal to infer the size and location of a tagged source, such as a tumor, within the animal. This is typically done using a single viewpoint and wavelength to create a 2D image of the light measured at the animal surface but suffers from inaccuracies due to poor localization and viewpoint dependence of light traveling through tissue. These can be improved via multiple viewpoints to allow 3D images to be created, however, source localization remains challenging due to the nonuniqueness of the problem; multiple source size/location give rise to the same single wavelength data. To add quantitative accuracy these 3D images can be used alongside optimization algorithms utilizing accurate models of light propagation to tomographically reconstruct the 3D spatial distribution of the bioluminescent source. Conventionally to achieve this a multimodal system is required to estimate the optical properties of the animal (e.g., DOT), and structural information (e.g., CT or MRI). Multispectral data are then required to overcome the non-uniqueness of the solutions as well as computational models to account for the path of light in free-space (animal to detector), increasing data collection and computational time. Advanced optimization algorithms use spectral derivative data, to allow accurate reconstructions whilst not requiring a free-space model, as light at similar wavelengths follow the same path. Finally, recent advancements in hardware and imaging systems have allowed quicker and cheaper collection of hyperspectral data, such as those based on compressive sensing.

An algorithm was developed with the aim of achieving better source localization by simultaneously calculating and updating the underlying optical properties and iteratively reconstructing the spatial light distribution, in 3D and shown to achieve good accuracy in simulated data and a tissue-mimicking block phantom.^22^ This algorithm has, for the first time been applied and validated in a set of *in-vivo* mouse models, including an orthotopic GBM model and two mice with self-luminous light source implanted in pancreas. Utilizing data from two different systems the algorithm has demonstrated robustness, further validating our phantom and theoretical studies. It is shown that using this optimization algorithm, localization accuracies of <1  mm is obtained on all cases, which is similar if not better than current “gold standard” methods that use literature published optical properties^18^ or using a different imaging modality.^4^ Application of this approach has shown that quantitative BLT is possible without the need for any prior knowledge about optical parameters, whilst achieving good localization accuracies. Simultaneously recovery of chromophore concentrations and optical parameters from the same dataset is possible, with no prior information, other than the spectral absorption spectrum of these absorbing molecules (oxy and deoxy hemoglobin), which can provide important biological information in a vast number of preclinical research settings such as radiation oncology, in guiding radiation and assessing treatment response for important orthotopic tumor models. This proposed and validated algorithm can pave the way for true molecular imaging of both exogenous and indigenous biological tumor characteristics and functionality such as hypoxia.

## Code, Data, and Materials Availability

The open-source NIRFAST toolbox was used to generate the model and to perform image reconstruction. The script used for all image reconstruction, based on NIRFAST, can be made available on request to the corresponding author.

## References

[1] Dehghani H. Davis S. C. Pogue B. W., “Spectrally resolved bioluminescence tomography using the reciprocity approach,” Med. Phys. 35(11), 4863–4871 (2008). MPHYA6 0094-2405 10.1118/1.2982138 19070220 PMC2737244

[2] Dehghani H. et al., “Near infrared optical tomography using NIRFAST: algorithm for numerical model and image reconstruction,” Commun. Numer. Methods Eng. 25(6), 711–732 (2008). CANMER 0748-8025 10.1002/cnm.1162 20182646 PMC2826796

[3] Dehghani H. et al., “Spectrally resolved bioluminescence optical tomography,” Opt. Lett. 31(3), 365–367 (2006). OPLEDP 0146-9592 10.1364/OL.31.000365 16480210

[4] Guggenheim J. A. et al., “Multi-modal molecular diffuse optical tomography system for small animal imaging,” Meas. Sci. Technol. 24(10), 105405 (2013). MSTCEP 0957-0233 10.1088/0957-0233/24/10/105405 24954977 PMC4061700

[5] Bentley A. Rowe J. E. Dehghani H., “Single pixel hyperspectral bioluminescence tomography based on compressive sensing,” Biomed. Opt. Express 10(11), 5549–5564 (2019). BOEICL 2156-7085 10.1364/BOE.10.005549 31799030 PMC6865106

[6] Alexandrakis G. Rannou F. R. Chatziioannou A. F., “Effect of optical property estimation accuracy on tomographic bioluminescence imaging: simulation of a combined optical-PET (OPET) system,” Phys. Med. Biol. 51(8), 2045–2053 (2006). PHMBA7 0031-9155 10.1088/0031-9155/51/8/006 16585844 PMC2997727

[7] Allard M. et al., “Combined magnetic resonance and bioluminescence imaging of live mice,” J. Biomed. Opt. 12(3), 034018 (2007). JBOPFO 1083-3668 10.1117/1.2745298 17614726

[8] Klose A. D. et al., “In vivo bioluminescence tomography with a blocking-off finite-difference SP3 method and MRI/CT coregistration,” Med. Phys. 37(1), 329–338 (2010). MPHYA6 0094-2405 10.1118/1.3273034 20175496 PMC2803718

[9] Jayet B. Morgan S. P. Dehghani H., “Incorporation of an ultrasound and model guided permissible region improves quantitative source recovery in bioluminescence tomography,” Biomed. Opt. Express 9(3), 1360–1374 (2018). BOEICL 2156-7085 10.1364/BOE.9.001360 29541527 PMC5846537

[10] Liu J. et al., “In vivo quantitative bioluminescence tomography using heterogeneous and homogeneous mouse models,” Opt. Express 18(12), 13102–13113 (2010). OPEXFF 1094-4087 10.1364/OE.18.013102 20588440 PMC2903618

[11] Naser M. A. Patterson M. S., “Algorithms for bioluminescence tomography incorporating anatomical information and reconstruction of tissue optical properties,” Biomed. Opt. Express 1(2), 512–526 (2010). BOEICL 2156-7085 10.1364/BOE.1.000512 21258486 PMC3017985

[12] Cong W. et al., “Practical reconstruction method for bioluminescence tomography,” Opt. Express 13(18), 6756–6771 (2005). OPEXFF 1094-4087 10.1364/OPEX.13.006756 19498692

[13] Feng J. et al., “An optimal permissible source region strategy for multispectral bioluminescence tomography,” Opt. Express 16(20), 15640–15654 (2008). OPEXFF 1094-4087 10.1364/OE.16.015640 18825203

[14] Balasubramaniam G. M. et al., “Tutorial on the use of deep learning in diffuse optical tomography,” Electronics 11(3), 305 (2022). ELECAD 0013-5070 10.3390/electronics11030305

[15] Feng J. C. et al., “Deep-learning based image reconstruction for MRI-guided near-infrared spectral tomography,” Optica 9(3), 264 (2022). 10.1364/OPTICA.446576 35340570 PMC8952193

[16] Yin L. et al., “Improved block sparse Bayesian learning method using K-nearest neighbor strategy for accurate tumor morphology reconstruction in bioluminescence tomography,” IEEE Trans. Biomed. Eng. 67(7), 2023–2032 (2020). IEBEAX 0018-9294 10.1109/TBME.2019.2953732 31751214

[17] Zhang X. et al., “Self-training strategy based on finite element method for adaptive bioluminescence tomography reconstruction,” IEEE Trans. Med. Imaging (2022). ITMID4 0278-0062 10.1109/TMI.2022.3167809 35436185

[18] Xu X. et al., “Quantitative bioluminescence tomography-guided conformal irradiation for pre-clinical radiation research,” Int. J. Radiat. Oncol. Biol. Phys. 111, 1310–1321 (2021). IOBPD3 0360-3016 10.1016/j.ijrobp.2021.08.010 34411639 PMC8602741

[19] Zhang B. et al., “Bioluminescence tomography-guided radiation therapy for preclinical research,” Int. J. Radiat. Oncol. Biol. Phys. 94(5), 1144–1153 (2016). IOBPD3 0360-3016 10.1016/j.ijrobp.2015.11.039 26876954 PMC4814325

[20] Deng Z. et al., “In vivo bioluminescence tomography center of mass-guided conformal irradiation,” Int. J. Radiat. Oncol. Biol. Phys. 106(3), 612–620 (2020). IOBPD3 0360-3016 10.1016/j.ijrobp.2019.11.003 31738948 PMC7007925

[21] Deng Z. et al., “In vivo bioluminescence tomography-guided radiation research platform for pancreatic cancer: an initial study using subcutaneous and orthotopic pancreatic tumor models,” Proc. SPIE 11224, 1122409 (2020). PSISDG 0277-786X 10.1117/12.2546503 PMC7677029 33223595

[22] Bentley A. Rowe J. E. Dehghani H., “Simultaneous diffuse optical and bioluminescence tomography to account for signal attenuation to improve source localization,” Biomed. Opt. Express 11(11), 6428–6444 (2020). BOEICL 2156-7085 10.1364/BOE.401671 33282499 PMC7687966

[23] Deng Z. et al., “Quantitative bioluminescence tomography for in vivo volumetric-guided radiotherapy,” Methods Mol. Biol. 2393, 701–731 (2022). 10.1007/978-1-0716-1803-5_38 34837208 PMC9098109

[24] Yang Y. et al., “Systematic calibration of an integrated x-ray and optical tomography system for preclinical radiation research,” Med. Phys. 42(4), 1710–1720 (2015). MPHYA6 0094-2405 10.1118/1.4914860 25832060 PMC4368593

[25] Zhang B. et al., “Multi-projection bioluminescence tomography guided system for small animal radiation research platform (SARRP),” Proc. SPIE 9701, 97010J (2016). PSISDG 0277-786X 10.1117/12.2211869

[26] Srinivasan S. et al., “Spectrally constrained chromophore and scattering near-infrared tomography provides quantitative and robust reconstruction,” Appl. Opt. 44(10), 1858–1869 (2005). APOPAI 0003-6935 10.1364/AO.44.001858 15813523

[27] Penrose R., “A generalized inverse for matrices,” Math. Proc. Cambridge Philos. Soc. 51(3), 406–413 (1955). MPCPCO 0305-0041 10.1017/S0305004100030401

[28] Dehghani H. et al., “Quantitative bioluminescence tomography using spectral derivative data,” Biomed. Opt. Express 9(9), 4163–4174 (2018). BOEICL 2156-7085 10.1364/BOE.9.004163 30615705 PMC6157772

[29] Basevi H. R. et al., “Compressive sensing based reconstruction in bioluminescence tomography improves image resolution and robustness to noise,” Biomed. Opt. Express 3(9), 2131–2141 (2012). BOEICL 2156-7085 10.1364/BOE.3.002131 23024907 PMC3447555

[30] Guggenheim J. A. et al., “Quantitative surface radiance mapping using multiview images of light-emitting turbid media,” J. Opt. Soc. Am. A Opt. Image Sci. Vis. 30(12), 2572–2584 (2013). 10.1364/JOSAA.30.002572 24323019

